# Electroacupuncture Improves Cognitive Function in Senescence-Accelerated P8 (SAMP8) Mice via the NLRP3/Caspase-1 Pathway

**DOI:** 10.1155/2020/8853720

**Published:** 2020-11-04

**Authors:** Zhitao Hou, Ruijin Qiu, Qingshuang Wei, Yitian Liu, Meng Wang, Tingting Mei, Yue Zhang, Liying Song, Xianming Shao, Hongcai Shang, Jing Chen, Zhongren Sun

**Affiliations:** ^1^College of Basic Medical and Sciences, Heilongjiang University of Chinese Medicine, Harbin, Heilongjiang 150040, China; ^2^Key Laboratory of Chinese Internal Medicine of the Ministry of Education, Dongzhimen Hospital Affiliated with Beijing University of Chinese Medicine, Beijing 100700, China; ^3^School of Acupuncture-Moxibustion and Tuina, Beijing University of Chinese Medicine, Beijing 100029, China; ^4^School of Acupuncture-Moxibustion and Tuina, Heilongjiang University of Chinese Medicine, Harbin, Heilongjiang 150010, China

## Abstract

*Background.* Clinically, electroacupuncture (EA) is the most common therapy for aging-related cognitive impairment (CI). However, the underlying pathomechanism remains unidentified. The aims of this study were to observe the effect of EA on cognitive function and explore the potential mechanism by which EA acts on the NLRP3/caspase-1 signaling pathway. *Main Methods*. Thirty male SAMP8 mice were randomly divided into the model, the 2 Hz EA and 10 Hz EA groups. Ten male SAMR1 mice were assigned to the control group. Cognitive function was assessed through the Morris water maze test. Hippocampal morphology and cell death were observed by HE and TUNEL staining, respectively. The serum IL-1*β*, IL-6, IL-18, and TNF-*α* levels were measured by ELISA. Hippocampal NLRP3, ASC, caspase-1, GSDM-D, IL-1*β*, IL-18, A*β*, and tau proteins were detected by Western blotting. *Key Findings*. Cognitive function, hippocampal morphology, and TUNEL-positive cell counts were improved by both EA frequencies. The serum IL-1*β*, IL-6, IL-18, and TNF-*α* levels were decreased by EA treatment. However, 10 Hz EA reduced the number of TUNEL-positive cells in the CA1 region and serum IL-1*β* and IL-6 levels more effectively than 2 Hz EA. NLRP3/caspase-1 pathway-related proteins were significantly downregulated by EA, but 2 Hz EA did not effectively reduce ASC protein expression. Interestingly, both EA frequencies failed to reduce the expression of A*β* and tau proteins. *Significance*. The effects of 10 Hz EA at the GV20 and ST36 acupoints on the NLRP3/caspase-1 signaling pathway may be a mechanism by which this treatment relieves aging-related CI in mice.

## 1. Introduction

Cognitive impairment (CI) is a common neurological disease among the elderly [1]. With the rapid aging of the global population, the proportion of patients with CI has been increasing year by year [2]. Current studies have found that the prevalence of dementia is 1% in people over 60 years old and more than 40% in people over 85 years old [3, 4]. Although the exact pathogenesis of CI is not yet clear, hippocampal pyroptosis induced by the chronic inflammatory cascade has been proposed by many scholars [5–7].

Pyroptosis is a new mechanism of cell death discovered in recent years. Caspase-1-mediated cell pyroptosis is a classical pathway that can be caused by chronic inflammation in aging [8]. Caspase-1-mediated cell pyroptosis is accompanied by the release of a large number of proinflammatory factors, which induces a cascade of amplified inflammatory responses, and staining reveals that the nuclear DNA undergoes changes similar to those that occur in cell apoptosis [9]. The major difference between caspase-1-mediated cell pyroptosis and cell apoptosis is that in the former process the cell membrane is destroyed, the cell swells due to increased permeability, and the contents of the cell are released to the extracellular environment [9, 10]. Furthermore, NOD-like receptor protein 3 (NLRP3), apoptosis-associated speck-like protein containing a CARD (ASC), and cysteinyl aspartate-specific protease-1 (caspase-1) are activated, forming the NLRP3 inflammasome [11]; additionally, the production of interleukin- (IL-) 1*β* and IL-18 is induced [12]. Then, the downstream signaling pathways are activated, promoting inflammation and inducing neural plasticity damage and other neuronal damage [13].

Electroacupuncture (EA) is commonly used as a clinical rehabilitation therapy to improve cognitive dysfunction. Two main EA frequencies are commonly used, namely, low frequency (2 Hz) and high frequency (10 Hz), both of which can effectively improve indexes of clinical outcomes [14]. EA can effectively reduce the levels of interleukin-1*β* (IL-1*β*), interleukin-6 (IL-6), interleukin-18 (IL-18), and tumor necrosis factor-*α* (TNF-*α*), helping to inhibit the inflammatory response in a variety of neurological diseases [15–17]. This study is aimed at revealing the potential mechanism by which different frequencies of EA improve cognitive function by inhibiting pyroptosis of the hippocampus in SAMP8 mice; the underlying goal is to provide new therapeutic ideas for a rational selection of EA therapy as an intervention in clinical and basic research on cognitive impairment.

## 2. Materials and Methods

### 2.1. Animals and Ethics Statement

Seven-month-old male senescence-accelerated P8 (SAMP8) mice and senescence-resistant R1 (SAMR1) mice were purchased from the experimental animal center of the First Affiliated Hospital of Tianjin University of Chinese Medicine (Tianjin, China). The experiment was conducted in accordance with the laboratory animal use regulations of the Animal Care and Use Committee of Heilongjiang University of Chinese Medicine. All animals were housed in a specific-pathogen-free room (20~22°C, 40%~60% humidity) under a 12 h day/night cycle with sterile feed and autoclaved water ad libitum. After 7 days of adaptation to the new environment, all animals underwent the formal experiment.

### 2.2. Animal Grouping and Administration

**Figure 1:**
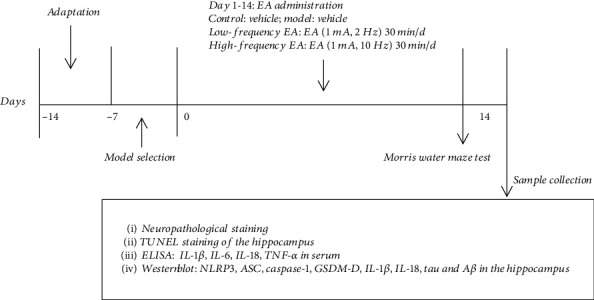
Experimental procedures.

Ten SAMR1 mice (7 months old, 22~25 g) were assigned to a control group (*n* = 10; day 0) that received no intervention. Thirty SAMP8 mice with CI (7 months old, 22~25 g) were randomly divided into three groups (*n* = 10 each; days 0, 1): a model group, which received no intervention; (2) a low-frequency EA group, which received EA (1 mA, 2 Hz) for 30 min once daily for 14 consecutive days; and (3) a high-frequency EA group, which received EA (1 mA, 10 Hz) for 30 min once daily for 14 consecutive days. Seven days before grouping and interventions, all mice underwent Morris water maze (Biobserve, Bonn, Germany) cued training tests to select for mice with CI. The criterion for selecting mice with CI was a significantly prolonged escape latency (>80 s) according to our previous study [18]. After the EA intervention ended, the mice were tested with the Morris water maze to evaluate the cognitive ability of each group. Samples of hippocampal tissue and serum were collected for further evaluation after cognitive function assessment. Neuropathological staining was performed by HE staining, and dead cells were stained with the terminal deoxynucleotidyl transferase-mediated dUTP nick-end labeling (TUNEL) method. Enzyme-linked immunosorbent assays (ELISAs) were used to detect the serum levels of IL-1*β*, IL-6, IL-18, and TNF-*α*. Western blotting was used to detect the expression levels of NLRP3, ASC, caspase-1, GSDM-D, IL-1*β*, IL-18, tau and A*β* (Figure 1).

### 2.3. EA Treatment

The mice from the two EA groups were fixed in a prone position with a stereotaxic device. The acupuncture needles (0.3 mm diameter, Guizhou Ande Medical Appliances, Ltd.) were inserted at a depth of 2-3 mm into the Baihui acupoint (GV20) and Zusanli acupoint (ST36), and then, a Great Wall Acupoint Nerve Stimulator (Model: KWD-808I, Changzhou Wujin Great Wall Medical Equipment Co., Ltd., Changzhou, China) was used. The control and model groups did not receive any EA treatment.

### 2.4. Cognitive Function Assessment

Cognitive function was assessed 7 days before EA administration by the Morris water maze test to select for mice with CI. The Morris water maze comprises two pieces of equipment: a circular pool and an automatic video analysis system for movement tracking and recording. The circular pool had a diameter of 200 cm and a height of 80 cm and was divided into four black quadrants. Different color stickers were placed above the inner walls of different quadrants. All animals performed 4 trials/day with 10 min intertrial intervals and a maximum trial duration of 90 s. Each mouse was allowed to remain on the platform for 30 s at the end of each trial, and a visible circular platform was placed in a different quadrant 1.5 cm above the water for each trial. The criterion for selecting mice with CI was a significantly prolonged escape latency (>80 s) before animal grouping and EA administration according to our previous study [18].

After EA administration ended, cognitive function was assessed in each group mice once per day for six consecutive days. This test was mainly divided into two aspects: (1) a navigation experiment conducted on the first five consecutive days to measure the learning ability of the mice during which each group of mice was placed into the water at a fixed position in the first quadrant, and the position and movement track of the mice in the water were recorded in real time with a high-speed camera. The assessment indexes were escape latency (seconds), the distance travelled (mm), speed (mm/s), and the time required for the mice to find and climb onto the platform. The maximum swimming time was set at 120 seconds. The time to reach the platform, travelling distance, and speed were observed and recorded. If an animal did not find the platform within 120 seconds, it was led to the platform, and the escape latency was recorded as 120 seconds. Then, the distance travelled and speed were recorded. (2) A space exploration experiment conducted on the 6^th^ day to measure the ability of the mice to maintain long-term memories during which the platform was removed, and the number of times that the mice crossed the location where the platform had been was calculated as the assessment index. During the experiment, all groups, including the control group, underwent the above tests. The laboratory environment was kept quiet, the water temperature was maintained at 22~26°C, and the light and objects around the water maze pool remained unchanged to reduce experimental errors caused by interference from the external environment. After each experiment, the pool was cleaned, the hair of the mice was dried, and the mice were given free access to food and water in accordance with the ethical requirements of animal welfare guidelines.

### 2.5. Neuropathological Staining

The mice were anesthetized with isoflurane gas anesthesia using a small animal anesthesia machine (Shanghai Sango Biotechnology Co., Ltd., Shanghai, China) after the final Morris water maze assessment. The brain tissues of the mice were placed in phosphoric acid buffer (PB) containing 4% paraformaldehyde and then fixed for 24 h. The tissues were embedded in paraffin and cut into continuous coronal sections. The slices were routinely dewaxed with dimethyl benzene and then rehydrated with the following alcohol series: xylene (I) 5 min, xylene (II) 5 min, anhydrous ethanol (I) 2 min, anhydrous ethanol (II) 2 min, 95% ethanol (I) 2 min, 95% ethanol (II) 2 min, and 80% ethanol 1 min. The slides were washed with distilled water for 1 min. Hematoxylin stain was applied for 5 min and rinsed away with tap water for 1 min. Next, 1% HCl ethanol was applied for 3 s for differentiation, and the samples were washed for 2 s; blue (with warm water or 1% ammonia, etc.) was applied for 10 s followed by a water rinse for 1 min, rinsing with distilled water for 1 min, and staining with 0.5% eosin for 2 min. Conventional dehydration, clearing, and sealing were performed. We selected 1 slice at intervals of 10 slices, the slice thickness was 4 *μ*m, and a total of 10 slices were selected for observation. Microscopy (OPTEC, BK-DM320/500, Germany) at 400, 800, and 1600x magnification was used to observe and photograph changes in the pathological structure of the hippocampal CA1 and CA3 regions.

### 2.6. TUNEL Staining

Paraffin sections of hippocampal tissues were washed with PBS, and the sections were placed in a DNase-free protease K solution of 20 g/mL and incubated at room temperature for 30 min. The specimens were washed with PBS for 5 min 3x, and then, 3% H_2_O_2_ solution was added for 10 min of room temperature incubation to eliminate endogenous peroxidase activity. The sections were permeabilized in PBS solution containing 0.1% Triton X-100 for 15 min. Then, a TUNEL detection solution containing TdT enzyme and biotin was drizzled onto the section and incubated in darkness at 37°C for 60 min. After the tissue was washed with PBS, the stop solution was applied dropwise to the sections, which were then incubated at room temperature for 10 min. The sections were washed with PBS for 5 min 3x; then, streptavidin-HRP working fluid was applied dropwise, and the sections were incubated at room temperature for 30 min. After 3 washes in PBS for 5 min each, DAB chromogen was applied dropwise to the sections, and they were incubated at room temperature for 10 min. The slices were washed with PBS for 5 min 3x, and nuclear staining was conducted with hematoxylin staining solution. Finally, the specimens were washed with PBS for 5 min 3x and sealed for observation. Five uncrossed and repeated fields were selected from each pathological section under an optical microscope, and cells with brown-yellow particles in the cytoplasm were regarded as positive apoptotic cells. We selected 1 slice at intervals of 10 slices, the slice thickness was 4 *μ*m, and a total of 10 slices were selected for observation. Ten fields per image were randomly selected to calculate the mean number of TUNEL-positive cells. ImagePro Plus 6.0 pathological image analysis software (Media Cybernetics, Inc., Rockville, MD, USA) was used to calculate the number of TUNEL-positive cells, and the average value was taken as the number of positive cells in the sample: TUNEL − positive cells ratio (%) = number of positive apoptotic cells/(number of positive apoptotic cells + number of negative apoptotic cells) × 100%.

### 2.7. ELISA

At the end of the experiment, eyeballs were removed for blood collection. The serum was separated by centrifugation at 3300 r/min at 4°C for 10 min, stored in a refrigerator at -80°C, and then used to measure the serum IL-1*β*, IL-6, IL-18, and TNF-*α* levels. Using an ELISA kit (Nanjing Jiancheng Bioengineering Institute, Nanjing, China), the IL-1*β*, IL-6, IL-18, and TNF-*α* levels were measured following the manufacturer's instructions.

### 2.8. Western Blotting

The frozen mouse hippocampal tissue in the -80°C refrigerator was taken out and thawed. The hippocampus was separated, transferred to an Eppendorf tube, and cut into pieces as much as possible with special scissors, and then, 50 mg of brain tissue was mixed with 300 *μ*L of RIPA lysate according to the instructions of the protein extraction reagent kit (Elabscience Biotechnology Co., Ltd., Wuhan, China). Then, 50 *μ*g of protein from each group was transferred to PVDF membranes (Thermo Fisher Scientific) and blocked with 5% nonfat milk overnight at 4°C. After washing, the membranes were incubated with the following primary antibodies: NLRP3 (rabbit polyclonal, 1 : 1000, Cell Signaling Technology, Danvers, MA, USA), ASC (rabbit polyclonal, 1 : 1000, Cell Signaling Technology, Danvers, MA, USA), GSDM-D (rabbit polyclonal, 1 : 1000, Cell Signaling Technology, Danvers, MA, USA), Caspase-1 (rabbit polyclonal, 1 : 1000, Cell Signaling Technology, Danvers, MA, USA), IL-1*β* (rabbit polyclonal, 1 : 1000, Cell Signaling Technology, Danvers, MA, USA), IL-18 (rabbit polyclonal, 1 : 1000, Cell Signaling Technology, Danvers, MA, USA), Tau (rabbit polyclonal, 1 : 1000, Cell Signaling Technology, Danvers, MA, USA), and A*β* (rabbit polyclonal, 1 : 1000, Cell Signaling Technology, Danvers, MA, USA). The membranes were subsequently sealed for incubation with the antibodies. AB luminescent solution (Beijing Priilet Co., Ltd., Beijing, China) was used for development and exposed onto the imaging system (Kodak, Rochester, NY, USA). Strip gray scale analysis was performed with ImageJ software (National Institutes of Health, Bethesda, MD, USA) and seven samples in each group.

### 2.9. Statistical Analysis

SPSS 22.0 (SPSS Inc. Chicago, IL, USA) and GraphPad Prism 8.0.1 (GraphPad Software Inc., San Diego, CA) software were used for statistics and mapping. Data were presented as the mean ± standard deviation. All data were collected and analyzed in a blinded manner. Using one-way analysis of variance (ANOVA), two-way ANOVA or two-way repeated-measures ANOVA followed by Bonferroni's post hoc test, we analyzed the escape latency, distance travelled, swimming speed, and target platform crossing number in the Morris water maze test. The Student-Newman-Keuls test was also used for multiple comparisons. Student's *t*-test (two-group comparison) was performed for intergroup comparisons under the condition of a normal distribution and homogeneity of variance. A nondifferential test was used when the variances were uneven. *P* < 0.05 was considered statistically significant.

## 3. Results

### 3.1. EA Treatment at the GV20 and ST36 Acupoints Improved Cognitive Function in SAMP8 Mice

**Figure 2:**
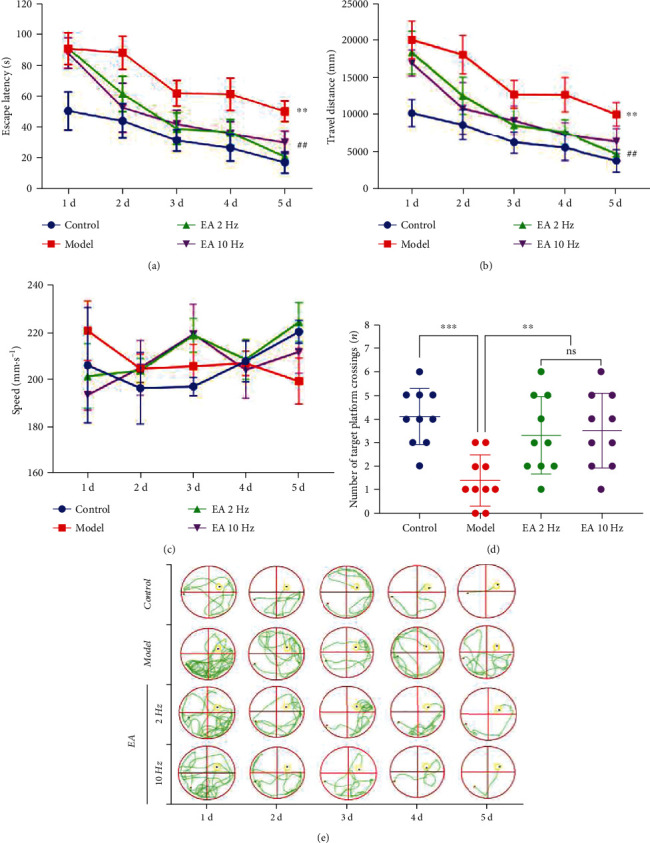
Electroacupuncture (EA) treatment enhanced learning and memory abilities in SAMP8 mice in two different cognitive function assessment experiments. (a) The escape latency in the navigation experiment. (b) The distance travelled in the navigation experiment. (c) The speed in the navigation experiment. (d) The target platform crossing number in the space exploration experiment. (e) The typical swimming trajectories in the navigation experiment. The data are shown as the mean ± SD of 10 mice per group. ^∗∗∗^*P* < 0.001 and ^∗∗^*P* < 0.01 vs. the model group; *^##^P* < 0.01 vs. the 2 Hz EA group. ns: not significant.

We applied two different frequencies of EA treatment to SAMP8 mice for 14 days to determine whether stimulation at the GV20 and ST36 acupoints can protect against cognitive dysfunction. Two aspects of cognitive function were assessed (Figures 2(a)–2(e)). Significantly prolonged escape latency and a longer distance travelled were observed for the SAMP8 mice in the model group compared with those in the control group (*P* < 0.01). The escape latency and distance travelled of the SAMP8 mice were significantly decreased after 14 days of EA treatment at both frequencies (*P* < 0.01). No direct evidence indicated the frequency at which EA more effectively reduced escape latency and the distance travelled (*P* > 0.05) (Figures 2(a) and 2(b)). Typical swimming trajectories of the mice in each group in the first five days are shown in Figure 1(e). During the experiment, no significant difference in swimming speed was observed between each group (*P* > 0.05) (Figure 2(c)), indicating that the test frequency did not cause exhaustion and that the motor function of the model animals was not damaged. On the other hand, we found that compared with the control group the number of target platform crossings in the model group decreased significantly (*P* < 0.01), and 14 days of consecutive EA treatment could effectively improve the crossing times of the SAMP8 mice. No direct evidence indicates the frequency at which EA was more effective (*P* > 0.05) (Figure 2(d)).

### 3.2. EA Treatment at the GV20 and ST36 Acupoints Alleviated Hippocampal Neuropathological Injury in SAMP8 Mice

**Figure 3:**
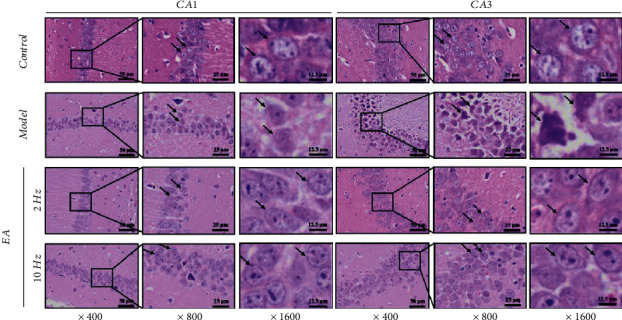
Electroacupuncture (EA) treatment was neuroprotective in the hippocampal CA1 and CA3 regions of SAMP8 mice. Magnification times, ×400, ×800, and ×1600. Bar, 50 *μ*m, 25 *μ*m, and 12.5 *μ*m.

HE staining showed that the neurons in the hippocampal CA1 and CA3 regions of SAMR1 mice in the control group had complete, clearly, and orderly structures, and no abnormalities were observed. In the model group, pyramidal cells in the hippocampal CA1 and CA3 regions were sparse, and the gaps increased. Obvious pathological, morphological, and structural changes (black arrow) were mainly observed: cell boundaries were unclear, cell body swelling increased, and nuclear shrinkage migrated. The pathological changes in neurons in the hippocampal CA1 and CA3 regions in the 2 Hz EA and 10 Hz EA groups were alleviated to a certain degree compared with those in the model group (black arrow), and the effects were the most significant in the 10 Hz EA group, with an orderly arrangement of neurons, clear cell boundaries, and a small number of morphological and structural abnormalities of neurons (black arrow) (Figure 3).

### 3.3. EA Treatment at the GV20 and ST36 Acupoints Reduced Cell Death in the Hippocampal Neurons of SAMP8 Mice

**Figure 4:**
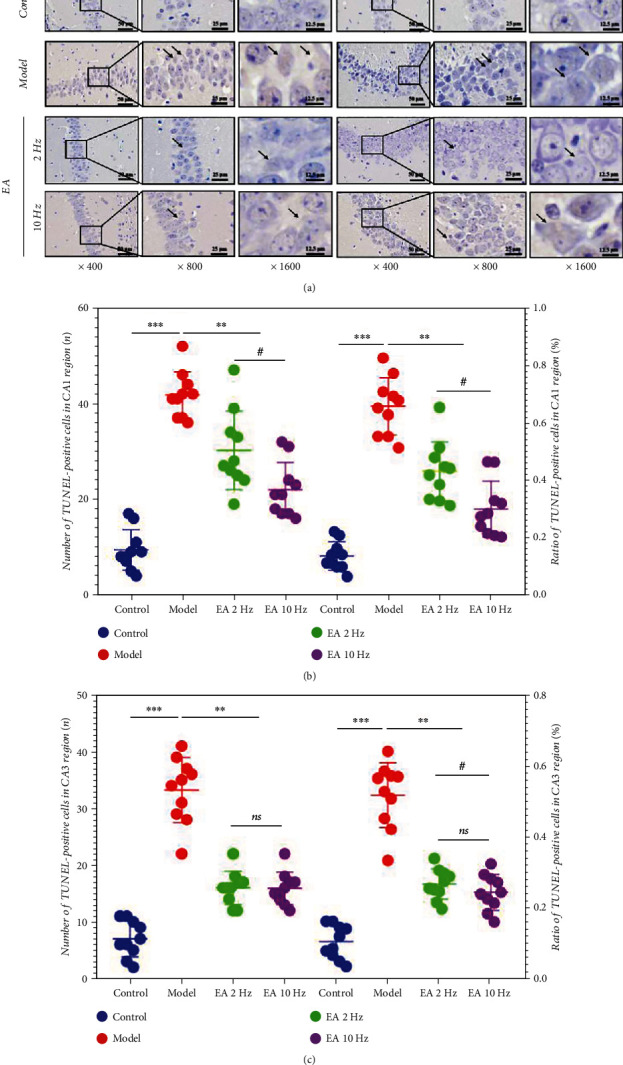
Electroacupuncture (EA) treatment limits neuronal cell death in the hippocampal CA1 and CA3 regions in SAMP8 mice. (a) Representative images of TUNEL staining in the hippocampal CA1 and CA3 regions from SAMR1 or SAMP8 mice. Magnification, ×400, ×800, and ×1600. Scale bars, 50 *μ*m, 25 *μ*m, and 12.5 *μ*m. (b) The number and ratio of TUNEL-positive cells in the CA1 region. (c) The number and ratio of TUNEL-positive cells in the CA3 region. Data are the mean ± SD (*n* = 10 per group). ^∗∗∗^*P* < 0.001 and ^∗∗^*P* < 0.01 vs. the model group; #*P* < 0.05 vs. the 2 Hz EA group. ns: not significant.

TUNEL-positive cells were identified by a brown-yellow lesion structure (black arrow) (Figure 4(a)). The number and ratio of TUNEL-positive neurons in the hippocampal CA1 and CA3 regions in the model group were significantly increased compared with those in the control group (Figures 4(b) and 4(c)) (*P* < 0.001), and the number and ratio of TUNEL-positive neurons in the hippocampal CA1 and CA3 regions in the 2 Hz EA and 10 Hz EA groups were significantly decreased compared with those in the model group (Figures 4(b) and 4(C)) (*P* < 0.01). Compared with EA at 2 Hz, EA at 10 Hz can effectively reduce the number and ratio of positive cells in the CA1 region (Figure 4(b)) (*P* < 0.05).

### 3.4. EA Treatment at the GV20 and ST36 Acupoints Decreased Serum Inflammatory Factor Levels in SAMP8 Mice

**Figure 5:**
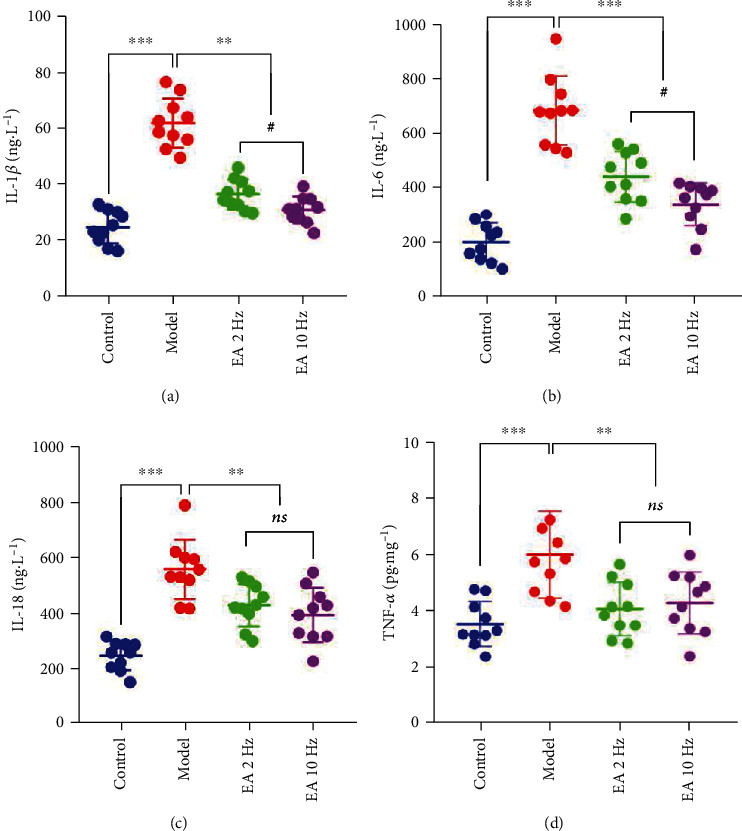
Electroacupuncture (EA) treatment attenuated the serum inflammatory factors in SAMP8 mice. (a) The level of interleukin-1*β* (IL-1*β*). (b) The level of interleukin-6 (IL-6). (c) The level of interleukin-18 (IL-18). (d) The level of tumor necrosis factor *α* (TNF-*α*). Data are presented as the means ± standard error of the mean (*n* = 10 per group). ^∗∗∗^*P* < 0.001 and ^∗∗^*P* < 0.01 vs. the model group; #*P* < 0.05 vs. the 2 Hz EA group. ns: not significant.

We detected the levels of four common inflammatory factors, IL-1*β*, IL-6, IL-18, and TNF-*α*, in the serum of mice and found that compared with the control group the levels of four inflammatory factors in the model group were significantly increased (*P* < 0.001). After 14 days of EA treatment, the levels of four inflammatory factors decreased significantly (*P* < 0.001 or *P* < 0.01) (Figures 5(a)–5(d)). Compared with 2 Hz EA therapy, 10 Hz EA significantly reduced the levels of IL-1*β* and IL-6 (*P* < 0.05) (Figures 5(a) and 5(b)).

### 3.5. EA Treatment at the GV20 and ST36 Acupoints Acted via the NLRP3/Caspase-1 Pathway to Improve Cognitive Function in SAMP8 Mice

**Figure 6:**
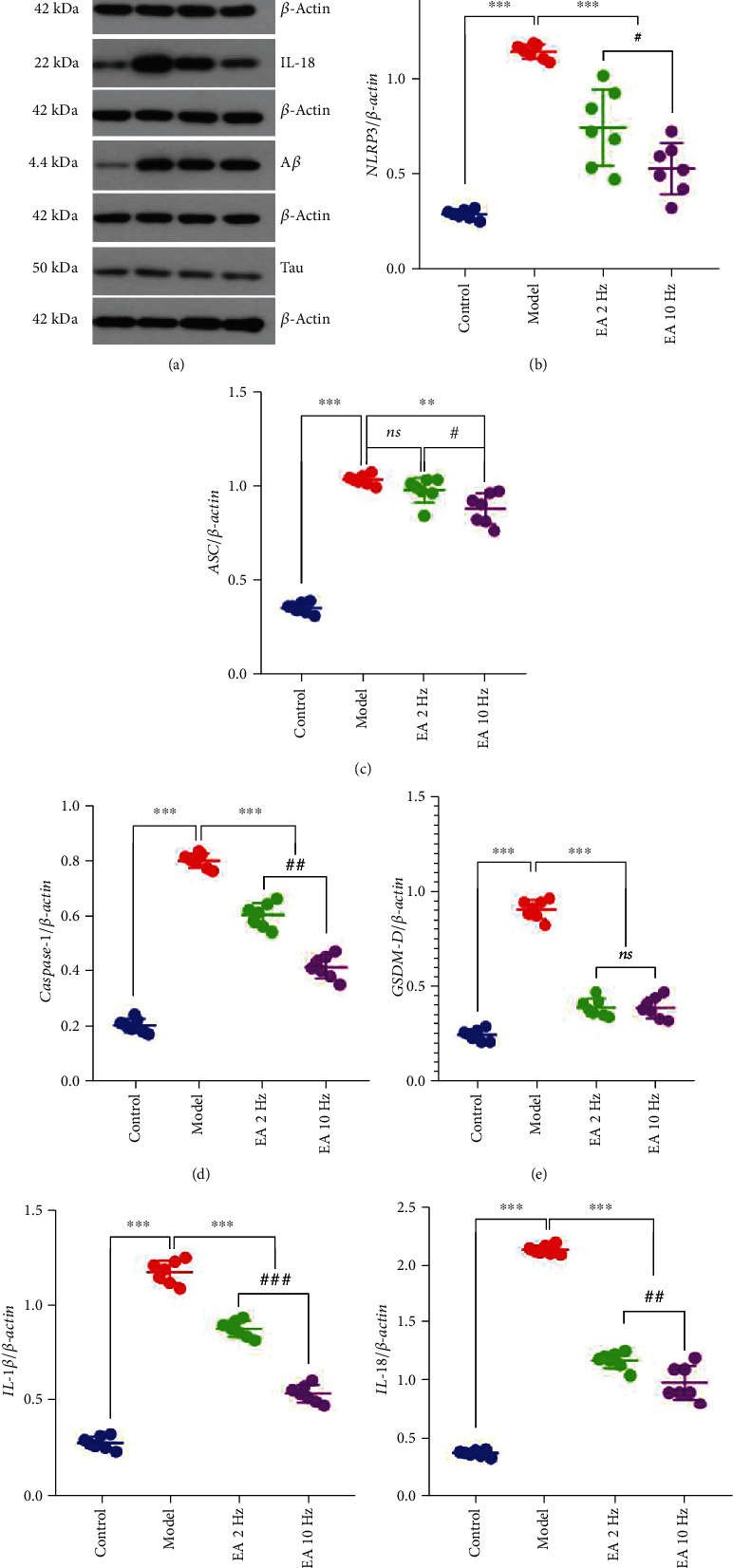
Expression of NLRP3/caspase-1 signaling-related proteins in the hippocampal tissue. (a) Western blot images showing the protein levels of NLRP3, ASC, caspase-1, GSDM-D, IL-1*β*, IL-18, A*β*, and tau in the hippocampus. (b–i) Quantification of NLRP3, ASC, caspase-1, GSDM-D, IL-1*β*, IL-18, A*β*, and tau bands in the hippocampus. Data are presented as the means ± standard error of the mean (*n* = 7 per group). ^∗∗∗^*P* < 0.001 vs. the model group; *^###^P* < 0.001 and *^##^P* < 0.01 vs. the 2 Hz EA group. ns: not significant.

We measured the expression of 8 proteins, including NLRP3, ASC, caspase-1, GSDM-D, IL-1*β*, IL-18, A*β*, and tau, in the hippocampal tissues of mice and found that compared with the control group the model group had increased expression of 7 proteins (all but tau; *P* < 0.001 for each comparison). Compared with the model group, the expression of 5 proteins, including NLRP3, caspase-1, GSDM-D, IL-1*β*, and IL-18, was significantly decreased after 14 days of 2 Hz EA treatment (*P* < 0.001). After 14 days of 10 Hz EA treatment, the expression of 6 proteins, including NLRP3, ASC, caspase-1, GSDM-D, IL-1*β*, and IL-18, was significantly decreased compared with the model group (*P* < 0.001). Compared with the 2 Hz EA group, the expression of 5 proteins, NLRP3, ASC, caspase-1, IL-1*β*, and IL-18, in the 10 Hz group was significantly downregulated (*P* < 0.05 or *P* < 0.01) (Figures 6(a)–6(i)). Interestingly, EA (2 Hz and 10 Hz) treatment had no significant effect on the expression of A*β* and tau proteins in the hippocampal tissues of SAMP8 mice, and the difference was not significant (*P* > 0.05) (Figures 6(a) and 6(h)–6(i)).

## 4. Discussion

The purpose of our study was to investigate whether SAMP8 mice with CI would benefit from different frequencies of EA treatment commonly used in clinical and previous studies [19, 20] and to explore the underlying mechanism based on NLRP3/caspase-1-mediated hippocampal pyroptosis induced by the chronic inflammatory cascade. The findings clearly suggest that EA treatment does have the hypothesized effect.

### 4.1. EA Is an Effective Therapy for Cognitive Impairment Caused by Aging

In recent years, a growing amount of evidence has confirmed EA as an effective therapy for a wide variety of diseases featuring cognitive dysfunction, such as Alzheimer's disease (AD) [21], vascular dementia (VD) [22], and CI [23, 24]. For example, EA treatment at the GV20 and ST36 acupoints improves model animal learning and memory abilities and protects against hippocampal injury, inhibits inflammatory factors, and regulates brain activity via antioxidative damage [25, 26]. An increasing number of countries have endorsed the efficacy and safety of EA treatment [27, 28]. These considerations led us to further explore the potential improving cognitive effects of EA treatment on CI in our SAMP8 mouse model.

In this study, we found that EA treatment at the GV20 and ST36 acupoints improved cognitive function in SAMP8 mice. According to our previous studies and other reports in the literature [18, 29], 7-month-old SAMP8 mice showed significant CI, and SAMR1 mice of the same age were selected as controls. Both animals are ideal models for studying CI induced by aging and are also ideal animal models for drug screening and treatment evaluation [30]. After 14 days of EA treatment, the learning ability of the animals in each group was investigated by navigation experiments for 5 consecutive days. EA at low (2 Hz) and high (10 Hz) frequencies effectively reduced the escape latency and travel distance of SAMP8 mice and resulted in clearer swimming tracks. Compared with the 2 Hz EA treatment, the 10 Hz EA treatment improved the learning ability of SAMP8 mice more significantly, but the difference was not significant (*P* > 0.05). On the sixth day, a spatial exploration experiment was conducted to investigate the memory ability of the animals in each group. EA at both frequencies effectively improved the crossing times of the target platform of SAMP8 mice, but no significant difference was found between the EA groups (*P* > 0.05). In the Morris water maze experiment, we did not observe significantly decreased swimming speed due to physical exhaustion and other factors during 6 consecutive days of testing, indicating that the Morris water maze is safe and reliable as a behavioral standard for evaluating the cognitive function of animals, and the experimental results will not be affected if each group is under the same experimental conditions. The above evidence suggests that EA treatment at either frequency can effectively improve the cognitive function of SAMP8 mice.

### 4.2. Increase in EA Frequency Plays a Better Role in Inhibiting Inflammation and Hippocampal Cell Death

The chronic inflammatory cascade reaction induced by aging is the main factor stimulating the death of hippocampal neurons. Our previous study found that IL-1*β*, IL-6, IL-18, and TNF-*α* were the inflammatory factors that were significantly increased in the serum of SAMP8 mice [18]. After 14 days of EA treatment, we found that both frequencies of EA treatment effectively reduced the four inflammatory factors. Compared with the 2 Hz EA treatment, the 10 Hz EA treatment has an advantage in reducing serum IL-1*β* and IL-6 levels. Cognitive function is critically related to the hippocampal CA1 and CA3 regions, which were observed in this study [18]. Compared with the hippocampus of SAMR1 mice, we found that those of SAMP8 mice exhibited a disordered pyramidal cell arrangement, incomplete membrane structure, shrinking nuclei, and other abnormal pathological findings, which was consistent with most previous reports using SAMP8 mice as model animals. More importantly, we found that EA, especially the 10 Hz EA treatment, can effectively improve these typical pathological changes, which are more obvious.

Hippocampal TUNEL staining and quantitative analysis are common detection methods conducted on the death of neurons. Compared with SAMR1 mice, SAMP8 mice were characterized by increased numbers and ratios of TUNEL-positive cells in the hippocampal CA1 and CA3 regions. The model mouse hippocampus may have neuronal cell death induced by inflammation, and this mode of cell death has been the focus area of CI in recent years. After 14 days of EA treatment, we found that EA could effectively reverse the cell death and the ratio of TUNEL-positive neurons in the hippocampal CA1 and CA3 regions and that 10 Hz EA treatment had a more significant reversal effect on the CA1 region than 2 Hz EA treatment. Furthermore, the increase in EA frequency may play a better role in inhibiting inflammation and hippocampal cell death.

### 4.3. High-Frequency EA Therapy Can Effectively Inhibit Hippocampal Pyroptosis through the NLRP3/Caspase-1 Pathway

At present, CI is believed to be mainly induced by abnormal changes in the tau protein framework, resulting in neurofibrillary tangles and the formation of senile plaques caused by excessive deposition of *β-*amyloid (A*β*) [31]. In addition, various in vivo and in vitro experiments have proven that pyroptosis is related to the pathogenesis of CI [5, 32], but the specific mechanism by which it participates remains unclear. The inflammatory response is a protective mechanism initiated by immune cells in response to injury- or infection-related factors; meanwhile, a long-term excessive inflammatory response may aggravate neural plasticity damage and disease progression. The neuroinflammatory response is generally believed to be regulated by NLRP3 inflammasome-dependent pyroptosis of neurons, and the death of neurons caused by pyroptosis is closely related to the onset of cognitive impairment [33].

The NLRP3 inflammasome comprises NLRP3, ASC, and caspase-1 [34]. GSDM-D (gasdermin-d) is the substrate of caspase-1 [35]. After GSDM-D protein is activated by caspase-1, it can cause cell membrane rupture, allowing water molecules and other substances to enter cells [36]; it can thereby induce the release of a large number of inflammatory cytokines, including IL-1*β* and IL-18, causing cell pyroptosis [36, 37]. Studies have shown that the NLRP3 inflammasome can identify A*β* and thus play an important role in the process [38]. Activated soluble interleukin-1 receptor type II, IL-18, and caspase-1 protein in cerebrospinal fluid (CSF) with mild CI and AD patients has been found by Lindberg et al. [39], indicating that inflammasome activation may be an important step in the development of early CI. Knocking out NLRP3 and caspase-1 in mouse models can largely prevent mice from developing CI-related learning and memory ability impairment and a serious pathological state [40]. Interestingly, a recent study showed that the absence of NLRP3 protected mice from aging-related inflammation and CI even in the brains of mice without excessive A*β* deposition [41]. Therefore, the inflammatory response dependent on NLRP3 is closely related to the cognitive decline associated with A*β* excessive deposition.

**Figure 7:**
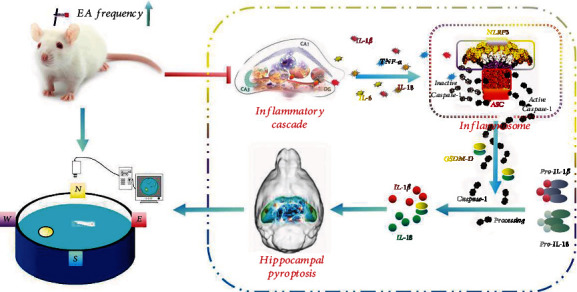
EA inhibited the activation of NLRP3 inflammasome to improve cognitive impairment. The attenuation of NLRP3 inflammasome activation ultimately reduced IL-1*β*, IL-18, and GSDM-D expressions and attenuated the inflammatory response of the hippocampus, thereby inhibiting hippocampal pyroptosis to improve cognitive impairment in SAMP8 mice.

We found that the expression of NLRP3/caspase-1 pathway-related 6 proteins and A*β* protein in the hippocampus was significantly increased in SAMP8 mice compared with SAMR1 mice. Interestingly, no significant difference was found in tau protein expression, consistent with previous studies showing that extensive deposition of A*β* protein was a typical pathological characteristic in the hippocampal tissues of SAMP8 mice. This evidence suggests that CI in SAMP8 mice is related to NLRP3/caspase-1 pathway-mediated pyroptosis and excessive deposition of A*β* protein. After 14 days of EA treatment, NLRP3/caspase-1 pathway-related proteins were significantly downregulated; the 2 Hz EA treatment did not effectively reduce the expression of ASC protein, a component of the NLRP3 inflammasome, but the 10 Hz EA treatment was effective. This suggests that higher EA frequencies are more effective in inhibiting NLRP3/caspase-1 pathway-mediated cell pyroptosis (Figure 7). In other words, increasing the EA frequency can effectively inhibit hippocampal pyroptosis under the condition of acupoint determination, and this finding has not been reported in the relevant literature yet. Our findings suggest that the frequency of EA therapy plays a crucial role in the treatment of CI, and the underlying mechanism of this phenomenon is that the inhibitory role of the inflammatory cascade-induced activation of NLRP3 inflammasome is enhanced with the increase in EA frequency within a reasonable range.

### 4.4. EA Could Not Reduce the A*β* and Tau Protein Expression of SAMP8 Mice and the Limitations of This Study

Interestingly, we found that the two common EA treatments failed to reduce the expression of A*β* and tau proteins in the hippocampal tissues of SAMP8 mice after 14 days. Due to the lack of high-quality reports on the mechanism of EA treatment on cognitive function in SAMP8 mice, analyzing the underlying causes is difficult. Notably, a recent report using EA treatment at the KI3 acupoint to intervene in 5XFAD mice found that EA can downregulate the expression level of A*β* protein [21]. We analyzed whether the failure to reduce the expression level of A*β* in this study may be related to acupoint selection. The reason why no significant change in tau protein was found may be related to the absence of such pathological characteristics in the SAMP8 model mice.

The limitations of this study are as follows. First, acupoint specificity is the main factor influencing acupuncture outcomes in clinical and animal studies. Whether EA at the GV20 and ST36 acupoints is the best choice or whether EA at other acupoints improves cognitive function in SAMP8 mice with CI through the restoration of the NLRP3/caspase-1 signaling pathway needs to be further explored. Second, acupuncture has bidirectional regulatory effects under different functional conditions; thus, a follow-up is necessary for conducting EA research on the changes in mouse electroencephalogram (EEG). Third, previous studies have found that pyroptosis occurs faster than other forms of cell death, such as apoptosis. The acupoint ST36 is on the leg, and the GV 20 acupoint is on the head. How the exact pathways that transduce the EA signal at two relatively distant acupoints to the hippocampus to affect protein translation in the animal model needs further exploration.

## 5. Conclusion

In conclusion, the present study provides evidence that EA treatment improves cognitive function, reduces inflammation, and inhibits pyroptosis in SAMP8 mice. The inhibition of NLRP3/caspase-1 signaling in the hippocampus may be involved in the beneficial effect of EA treatment on cognitive function.

## Data Availability

The data used to support the findings of this study are available from the corresponding author upon request.
